# Effects of different geriatric nursing teaching methods on nursing students’ knowledge and attitude: Systematic review and network meta-analysis

**DOI:** 10.1371/journal.pone.0300618

**Published:** 2024-05-31

**Authors:** Yifen Cheng, Shuqin Sun, Yu Hu, Jing Wang, Wenzhen Chen, Yukuan Miao, Hui Wang

**Affiliations:** 1 Department of Orthopedics, The First Affiliated Hospital of Anhui University of Traditional Chinese Medicine, Hefei, Anhui, China; 2 School of Nursing, Anhui University of Traditional Chinese Medicine, Hefei, Anhui, China; 3 Department of Endocrinology, The First Affiliated Hospital of Anhui University of Traditional Chinese Medicine, Hefei, Anhui, China; 4 School of Nursing, Chuxiong Medical College, Chuxiong, Yunnan, China; Murcia University, Spain, SPAIN

## Abstract

**Objective:**

The purpose of this study was to evaluate the effect of different teaching methods of geriatric nursing on the mastery of geriatric knowledge among nursing students and their attitude toward the elderly.

**Methods:**

Relevant randomized controlled trials (RCTs) and quasi-experimental studies on teaching methods to improve nursing students’ knowledge and attitude were systematically retrieved in electronic databases. The time scale of retrieval spans from the database establishment to January 2024, and the database consists of PubMed, the Cochrane Library, Web of Science, Embase, China National Knowledge Infrastructure Database (CNKI), China Biological literature database (CBM), Wanfang Database and VIP Database. Network meta-analysis was performed by Stata 16.0 software.

**Results:**

Thirty-nine studies involving 5310 nursing students met our inclusion criteria, and a total of 6 teaching methods were analyzed. According to the surface under the cumulative ranking (SUCRA) ranking, problem-based learning (PBL) was most effective in enhancing the knowledge mastery of geriatric nursing, while simulation-based learning (SBL) demonstrated the best application effect in improving nursing students’ attitude toward the elderly. When considering both knowledge acquisition and attitude improvement simultaneously, service learning combined with traditional teaching method (SL+TTM) was found to exhibit the most optimal effectiveness.

**Conclusion:**

Educators in geriatric nursing education should prioritize the adoption of PBL, SBL and SL + TTM to enhance nursing students’ knowledge and attitude.

**Protocol registry:**

PROSPERO (CRD42023442001).

## Introduction

With the global elderly population continuously increasing, China has become the country with the largest middle-aged and elderly population. The result of the seventh national census showed that the number of individuals aged 60 and above in China reached 264.0188 million in 2020, accounting for 18.70% of the total population, and the process of population aging was on the rise [1]. By the end of 2019, the prevalence of chronic diseases among individuals aged 60 and above in China had reached 69.13% [2], signaling a huge demand for elderly care professionals. Nursing students constitute a pivotal cohort in the provision of nursing services for elderly patients. Their mastery of geriatric knowledge and attitude toward the elderly will directly shape the extent to which caregivers’ willingness to provide elderly care [3] and their vocational preferences [4]. The positive attitude among nursing students toward the elderly has the potential to enhance the quality of geriatric care. Adopting a negative attitude, however, can lead to substantial adverse ramifications, exacerbating societal issues including discrimination and instances of elder mistreatment [5]. Research findings indicate that most nursing students lack knowledge in geriatric nursing, hold age-discriminatory attitude, and still need improvement [6]. Geriatric nursing education plays a vital role in shaping nursing students’ perceptions of aging, and the instructional design of geriatric nursing courses can enhance nursing students’ knowledge and positive attitude toward the elderly [7, 8].

Traditional teaching methods (TTM) involve instructors actively lecturing on theoretical knowledge in a relatively uniform manner [9], which may hinder nursing students from taking an active and enthusiastic approach toward geriatric nursing knowledge, consequently failing to significantly improve their positive attitude toward serving the elderly. In recent years, nursing educators have recognized the importance of improving nursing students’ knowledge mastery and their attitude toward the elderly. As a result, new teaching methods have been devised and substantiated with distinct applied advantages within geriatric nursing education, such as simulation-based learning (SBL), service learning combined with traditional teaching method (SL+TTM), problem-based learning (PBL), and learning with older people programme (LOPP). However, the comparative study of these teaching methods on the impact of knowledge and attitude of nursing students about the elderly is limited, making it imperative to assess and compare the efficacy of different teaching methods. Traditional Meta-analysis cannot compare the effects of different interventions, thus fails in choosing the best teaching method for elderly nursing.

This study employs a network meta-analysis to comparatively assess the effects of six distinct geriatric nursing instructional methods on the enhancement of nursing students’ knowledge and attitude toward the elderly. The findings offer valuable insights for informing the selection of geriatric nursing teaching paradigms.

## Materials and methods

### Search strategy

The systematic review adhered to the PRISMA-NMA guidelines during its execution and reporting (shown in S1 Table). PubMed, The Cochrane Library, Web of Science, Embase, China National Knowledge Infrastructure (CNKI), China Biology Medicine (CBM), VIP and Wanfang databases were systematically searched from the inception of these databases up to January 2024. A combination of subject terms and free-text terms were used in the search strategies, as follows: (nursing students or Students, Nursing or Nursing Student or Pupil Nurses or Nurses, Pupil or Nurse, Pupil or Pupil Nurse) AND (Geriatric nursing or gerontology or Geriatric or curriculum of gerontological nursing or gerontological nursing education or education) AND (simulation-based learning or SBL or simulation-based education or SBE or simulation or experiential learning or experiential teaching or service learning or problem-based learning or PBL or lecture-based learning or LBL or Learning with older people programme or LOPP or traditional teaching method) AND [knowledge or final examination scores or final course scores or test scores or school marks or scholastic achievement or theoretical knowledge or academic performance or course grade or exam scores or final exam scores or attitude* or attitude(s) toward(s)/to older/geriatric/old/elderly/aged/aging/seniors/geriatrics], and trace has been added into the literature references do. The initial search strategy for PubMed is shown in S2 Table.

### Inclusion and exclusion criteria

#### Inclusion criteria

① Study design: Randomized Controlled Trials (RCTs) or quasi-experimental studies; ② Participants: Nursing students receiving geriatric nursing education; ③ Intervention: Comparisons of novel teaching methods with placebo simulation-based learning or traditional teaching method; novel teaching methods: Simulation-based learning (SBL), Service learning + traditional teaching method (SL+TTM), Problem-based learning (PBL), and Learning with older people programme (LOPP); ④ Outcomes: Nursing students’ knowledge mastery and their attitude toward the elderly, and no restriction on the measurement types or evaluation methods of nursing students’ knowledge and attitude is applied.

#### Exclusion criteria

① Single arm trials, Self-control studies or mixed method studies; ② Interventions that do not include the above teaching methods; ③ Reviews, meta-analysis, conference abstracts, studies with non-full text and duplicate publications; ④ Lack of data on knowledge and attitude toward the elderly.

### Literature screening and data extraction

Two nursing postgraduate students will read the title, abstract and full text of the literature according to the inclusion and exclusion criteria for independent screening, data extraction and cross-checking. The disputes will be resolved through expert discussion or consultation with the tutor. The extraction content mainly includes: first author, source of research objects, sample size, age, gender, education background, intervention measures, Outcomes and measuring tool, and research type.

### Quality assessment

The included RCTs were independently evaluated according to the 5 aspects of the revised Cochrane Collaboration’s Risk of Bias 2 (RoB2) tool [10], and the 7 aspects of the Risk of Bias in Non-randomized studies of interventions (ROBINS-I) tool [11] were used to independently evaluate the included quasi-experimental studies. The bias risk for each RCT was categorized as low, some concerns, or high, whereas for each quasi-experimental study, it was categorized as low, moderate, serious, or critical. Each study assessment was conducted independently by two nursing master’s students. Disputes regarding the results were resolved through expert discussion or consultation with the tutor. Finally, risk of bias VISualization (robvis) tool [12] was used to create the figures.

### Statistical methods

Stata16.0 software was used for data analysis and graph drawing. Standardized mean difference (SMD) and 95% confidence interval (95% CI) were used for comparative analysis of measurement data. The results of network meta-analysis were presented through forest maps, and the application effects of each method were compared by the surface under the cumulative ranking (SUCRA) graph. A comprehensive evaluation of teaching methods ranking is presented by drawing a scatter plot based on the SUCRA values obtained by each intervention in terms of both knowledge and skill attitude. The node analysis method is used to test the inconsistency. If the direct comparison and the indirect comparison P > 0.05, the consistency model can be adopted. Publication bias was assessed using funnel plots and Egger’s test.

## Results

### Literature screening results and basic characteristics

**Fig 1:**
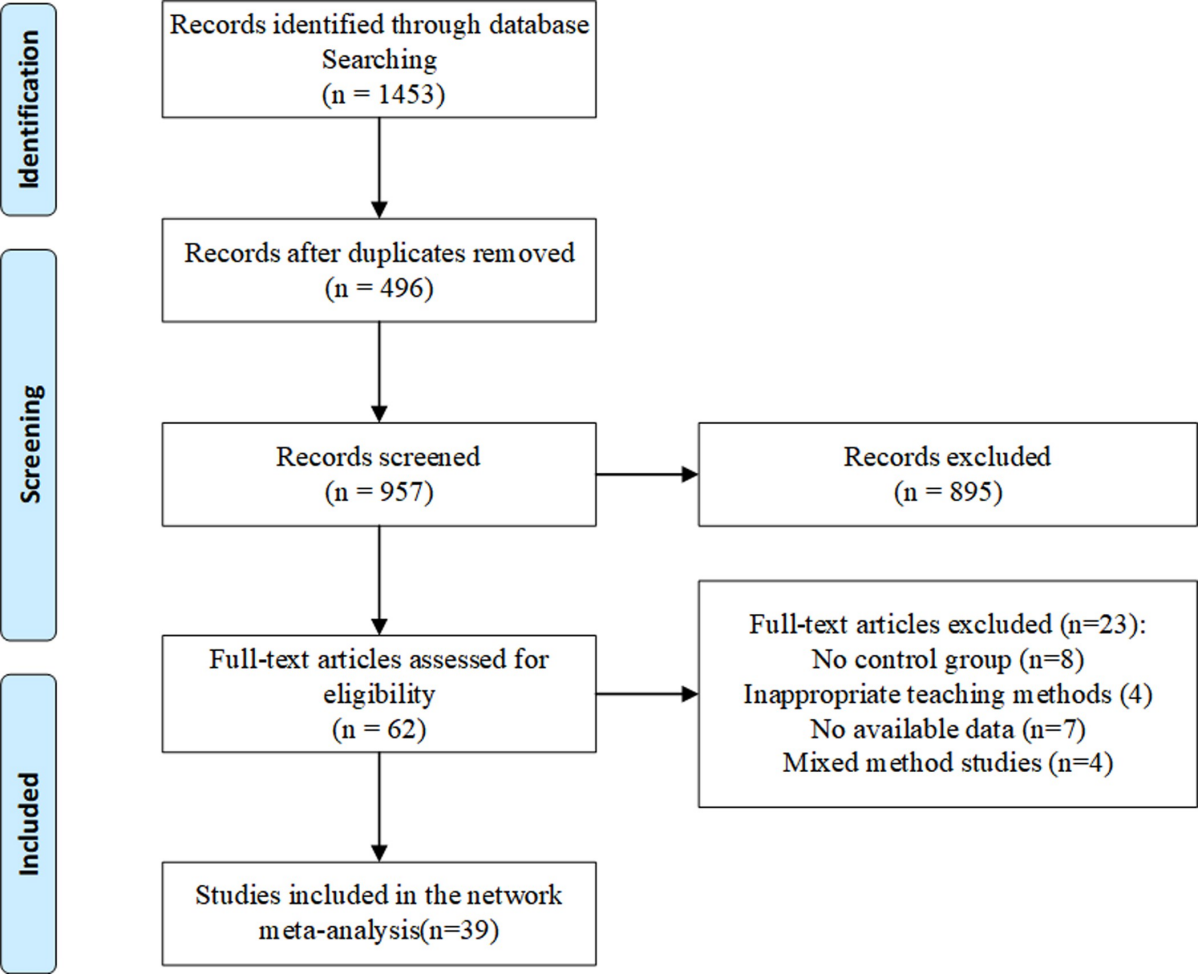
Flow diagram of study selection.

A total of 1453 articles were retrieved for the first time, and 62 articles were left after re-screening, excluding articles that did not meet the inclusion criteria. And 39 literatures were finally included based on further reading the full text. The whole selection process is shown in Fig 1. The included literatures were from China, Turkey, Iran, Finland with a total sample size of 5310 nursing students. The basic characteristics are shown in Table 1.

**Table 1:** Characteristics of included studies.

Author (Year)	Country	Sample size (T/C)	Age (years, $\bar{x}\pm S$)	Gender (male/female)	Educational background	Intervention	Outcomes and measuring tool	Study design
Li et al. 2020 [13]	China	51/51	NR	NR	Junior college	T:SL+TTM C:TTM	Knowledge(exam),attitude(KAOP)	RCT
Song et al. 2017 [14]	China	150/150	20∼25	T:14/136 C:12/138	Undergraduate/Junior college	T:SL+TTM C:TTM	Knowledge(exam), attitude(KAOP)	RCT
Pekcetin et al. 2021 [15]	Turkey	27/28	T:20.11±1.25 C:20.48±1.15	T:9/18 C:11/17	Undergraduate	T:SL+TTM C:TTM	Attitude(KAOP)	RCT
Liu et al. 2019 [16]	China	58/61	T:17∼22 C:18∼22	T:8/50 C:9/53	Junior college	T:SL+TTM C:TTM	Knowledge(FAQ), attitude(KAOP)	RCT
Cheng et al. 2020 [17]	China	69/70	T: 60<23years, 9≥23years C:66<23years, 4≥23years	T:20/49 C:14/56	Undergraduate	T:SBL C:Placebo SBL	Attitude(KAOP)	RCT
Yu et al. 2022 [18]	China	72/71	NR	NR	Junior college	T:SL+TTM C:TTM	Knowledge(exam), attitude(KAOP)	RCT
Wang et al. 2020 [19]	China	79/83	NR	T:9/70 C:11/72	Junior college	T:SBL C:TTM	Knowledge(exam), attitude(KAOP)	Quasi-experimental study
Demirtas et al. 2021 [20]	Turkey	59/60	T:20.42±3.48 C:20.96±3.01	T:7/52 C:8/52	Undergraduate	T:SBL C:TTM	Attitude(KAOP)	Quasi-experimental study
Mandegari Bamakan et al. 2021 [21]	Iran	35/35	T:20.85±1.16 C:21.25±2.20	T:16/19 C:15/20	Undergraduate	T:SBL C:TTM	Knowledge(FAQ), attitude(KAOP)	Quasi-experimental study
Koskinen et al. 2016 [22]	Finland	46/41	T:23.6±4.8 C:23.4±4.2	T:3/43 C:5/36	Undergraduate	T:LOPP C:TTM	Knowledge(FAQ), attitude(KAOP)	Quasi-experimental study
Torkshavand et al. 2020 [23]	Iran	35/35	T:23.86±3.68 C:23.40±2.89	T:16/19 C:15/20	Undergraduate	T:SBL C:TTM	Knowledge(Questionnair), attitude(UCLA-GAS)	Quasi-experimental study
Wu et al. 2022 [24]	China	64/62	T:18.6±0.07 C:19.1±0.07	T:4/60 C:23/59	NR	T:PBL C:TTM	Knowledge(GPN-K), attitude(GPN-A)	Quasi-experimental study
Wu et al. 2022 [25]	China	62/62	T:18∼20 C:18∼21	T:9/53 C:11/51	Undergraduate	T:SL+TTM C:TTM	Knowledge(FAQ), attitude(KAOP)	RCT
Cai et al. 2014 [26]	China	180/180	T:20.83±1.87 C:20.72±1.99	T:12/168 C:10/170	Junior college	T:SBL C:TTM	Knowledge (exam)	RCT
Zheng et al. 2017 [27]	China	35/35	T:18.4±0.5 C:18.2±0.6	T:1/34 C:2/33	Junior college	T:PBL C:TTM	Knowledge (exam)	RCT
Liu et al. 2009 [28]	China	60/60	NR	NR	Junior college	T:PBL C:TTM	Knowledge (exam)	RCT
Zhang et al. 2011 [29]	China	50/51	18∼22	5/96	Junior college	T:PBL C:TTM	Knowledge (exam)	RCT
Li et al. 2008 [30]	China	40/38	NR	NR	Junior college	T:PBL C:TTM	Knowledge (exam)	RCT
Sun et al. 2010 [31]	China	56/57	T:21±2.0 C:21±2.0	T:3/53 C:4/53	Junior college	T:SBL C:TTM	Knowledge (exam)	RCT
Wang et al. 2014 [32]	China	113/119	18∼21(19.31±0.15)	0/232	Junior college	T:SBL C:TTM	Knowledge (exam)	RCT
Ao et al. 2020 [33]	China	60/60	T:20.44±0.39 C:19.24±0.77	T:4/56 C:5/55	Junior college	T:PBL C:TTM	Knowledge (exam)	RCT
He et al. 2016 [34]	China	39/39	NR	NR	Junior college	T:SBL C:TTM	Knowledge (exam)	RCT
Yang 2015 [35]	China	99/102	19∼22	T:3/96 C:2/100	Junior college	T:SBL C:TTM	Knowledge (exam)	RCT
Guo et al. 2020 [36]	China	83/126	T:17∼20 C:17∼21	T:5/78 C:11/115	Junior college	T:SBL C:TTM	Knowledge (exam)	RCT
Guo et al. 2018 [37]	China	50/50	T:20.29±0.92 C:20.28±0.85	T:10/40 C:15/35	Junior college	T:PBL C:TTM	Knowledge (exam)	RCT
Li 2021 [38]	China	100/100	T:20.48±1.04 C:20.31±1.01	T:8/92 C:11/89	Junior college	T:SBL C:TTM	Knowledge (exam)	RCT
Chi et al. 2020 [39]	China	50/50	T:23.01±0.52 C:22.03±0.49	T:4/46 C:2/48	NR	T:PBL C:TTM	Knowledge (exam)	RCT
Zhou 2021 [40]	China	34/34	T:28.4±3.2 C:28.6±3.1	T:9/25 C:10/24	NR	T:SBL C:TTM	Knowledge (exam)	RCT
Cen et al. 2018 [41]	China	100/100	T:21.53±0.51 C:21.49±0.47	T:6/94 C:6/94	Undergraduate/Junior college	T:PBL C:TTM	Knowledge (exam)	RCT
Su 2018 [42]	China	45/45	T:19.7±2.4 C:19.8±2.5	T:0/45 C:0/45	Junior college	T:SBL C:TTM	Knowledge (exam)	RCT
Xia et al. 2016 [43]	China	40/40	17∼23	8/82	Undergraduate/Junior college/ Secondary vocational school	T:PBL C:TTM	Knowledge (exam)	RCT
Tang 2019 [44]	China	50/50	T:19∼23 C:18∼23	T:10/40 C:12/38	NR	T:PBL C:TTM	Knowledge (exam)	RCT
Wu et al. 2019 [45]	China	42/42	T:18∼24 C:17∼24	T:1/41 C:2/40	NR	T:PBL C:TTM	Knowledge (exam)	RCT
Feng 2022 [46]	China	80/82	T:16.49±0.62 C:16.39±0.56	T:22/58 C:20/62	Secondary vocational school	T:SBL C:TTM	Knowledge (exam)	Quasi-experimental study
Ma 2020 [47]	China	45/45	T:21.13±1.03 C:21.56±1.02	T:5/40 C:4/41	Junior college	T:PBL C:TTM	Knowledge (exam)	Quasi-experimental study
Wu et al. 2018 [48]	China	110/110	T:21.05±0.48 C:21.12±0.53	T:19/91 C:20/90	Undergraduate	T:SBL C:TTM	Knowledge (exam)	Quasi-experimental study
Wang et al. 2015 [49]	China	27/25	T:21.96±0.81 C:21.80±0.71	T:0/27 C:0/25	Undergraduate	T:PBL C:TTM	Knowledge (exam)	Quasi-experimental study
Zhang et al. 2022 [50]	China	56/60	T:21.96±0.81 C:20.80±0.71	T:7/49 C:9/51	Junior college	T:PBL C:TTM	Knowledge (exam)	Quasi-experimental study
Zhang et al. 2015 [51]	China	180/180	18∼22	T:8/172 C:9/172	Junior college	T:SBL C:TTM	Knowledge (exam)	Quasi-experimental study

Notes: NR (not reported); T (treatment group), C (control group); KAOP (kogan’ s attitude toward old people scale); FAQ (palmore facts on aging quiz scale); UCLA-GAS (the university of california at los angeles geriatrics attitudes scale); GPN-A (geropsychiatric psychological symptoms and health problem nursing attitude scale); GPN-K (geropsychiatric psychological symptoms and health problem nursing knowledge scale).

### Quality of studies

**Fig 2:**
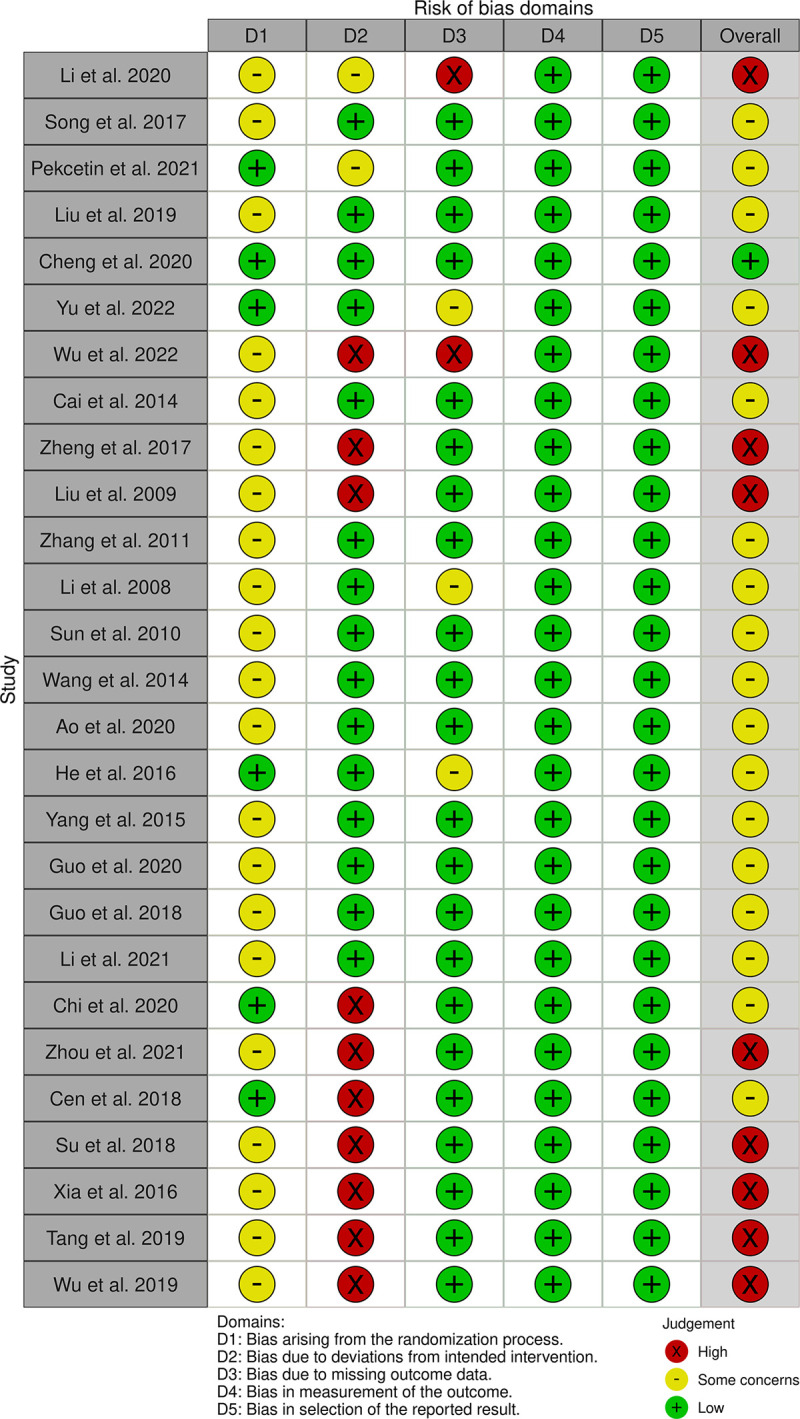
Risk of bias in RCTs.

**Fig 3:**
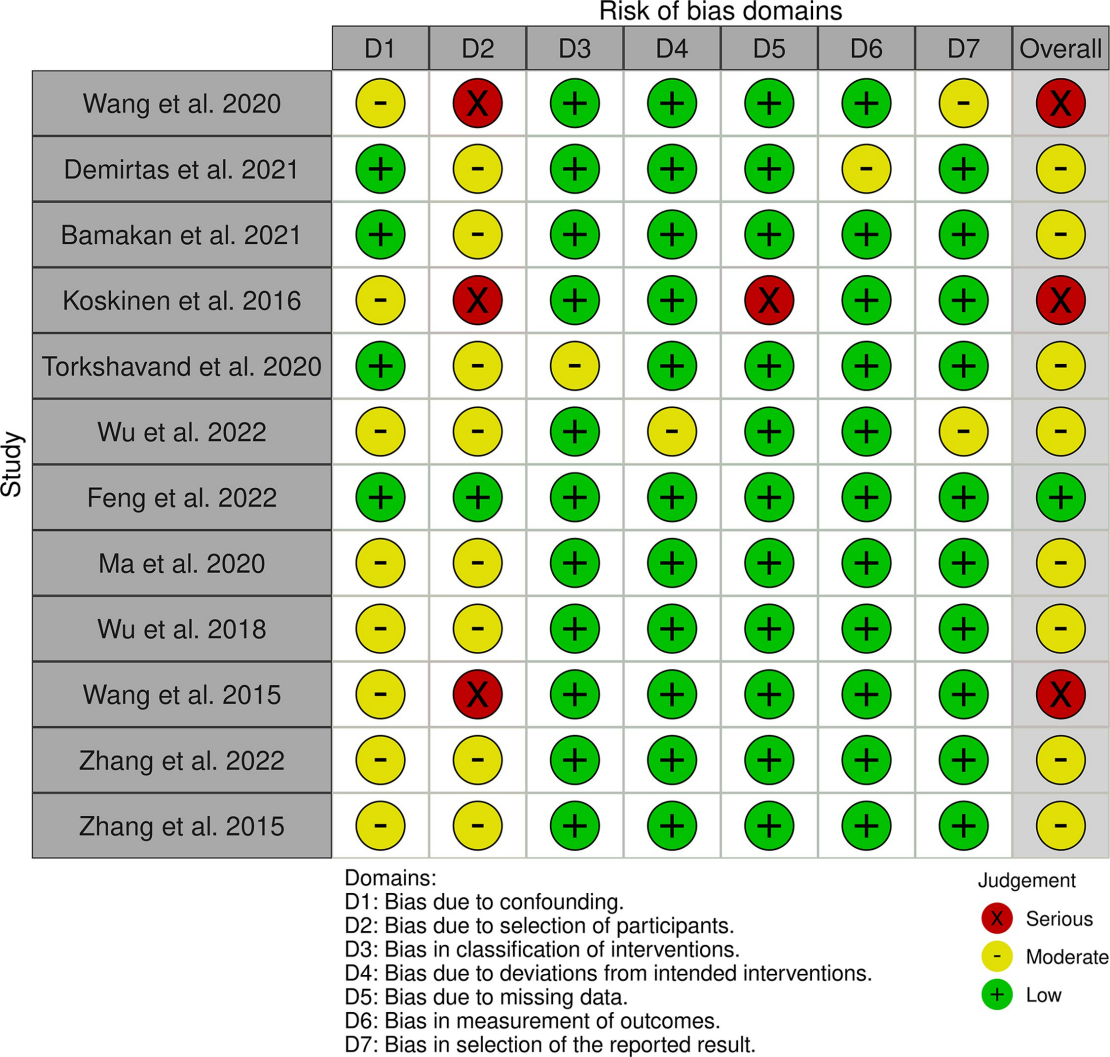
Risk of bias in quasi-experimental studies.

According to the RoB2 tool, among the 27 RCTs, one has an overall bias risk score categorized as "low risk," 17 as "some concerns," and 9 are categorized as "high risk," as illustrated in Fig 2. Regarding the overall bias risk in non-randomized controlled trials (ROBINS-I), among the 12 quasi-experimental studies, 1 is rated as "low risk," 8 as "moderate bias risk," and 3 are rated as "serious bias risk," as illustrated in Fig 3.

### Results of network meta-analysis

#### Network diagram

**Fig 4:**
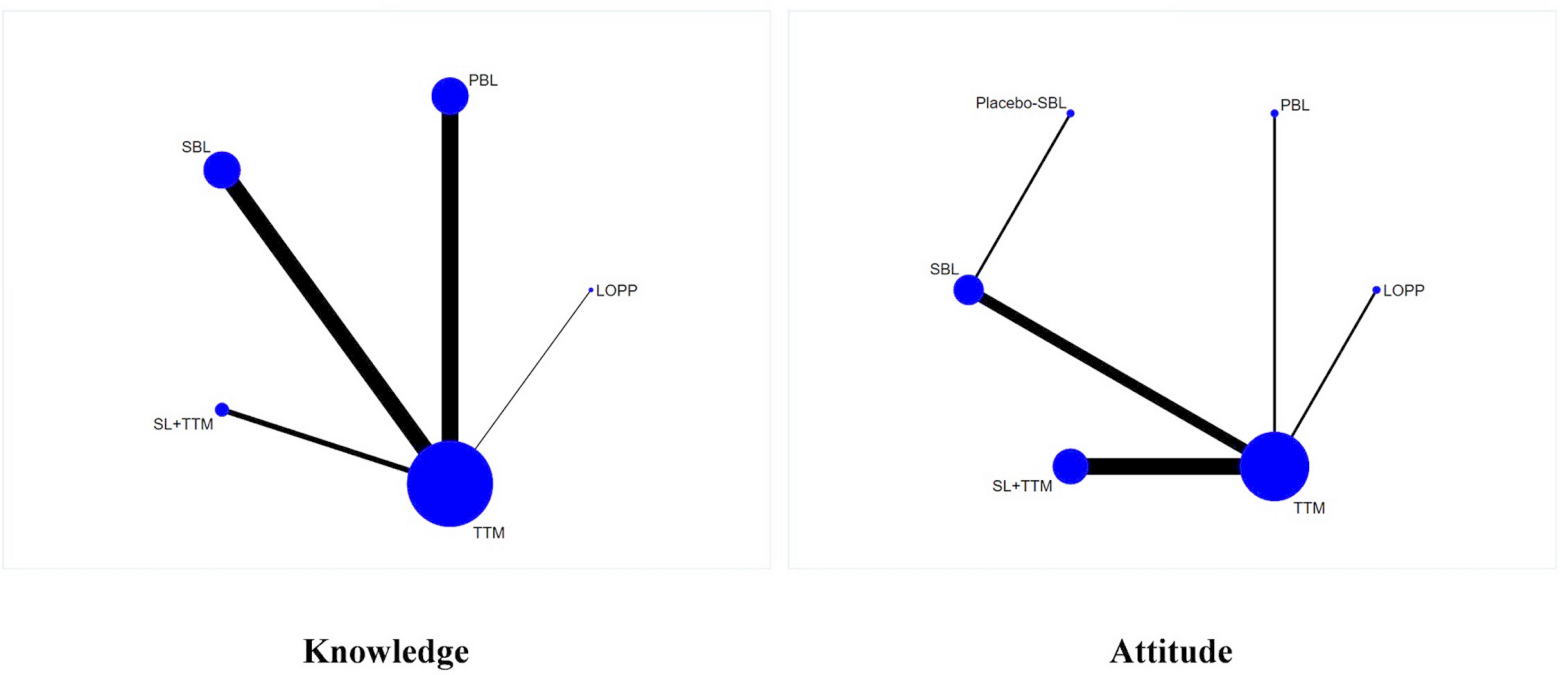
Network plot of the influence of different teaching methods on nursing students’ knowledge and attitude.

The network depiction illustrating the impact of diverse teaching methods on nursing students’ knowledge and their attitude toward the elderly is presented in Fig 4. Each teaching method is represented by a distinct data point, with the size corresponding to the level of acceptance among nursing students regarding the intervention of the given teaching method. Furthermore, the thickness of the solid lines connecting the data points reflects the extent of direct comparative research conducted between the respective pairs of teaching methodologies.

#### Network meta-analysis.

**Fig 5:**
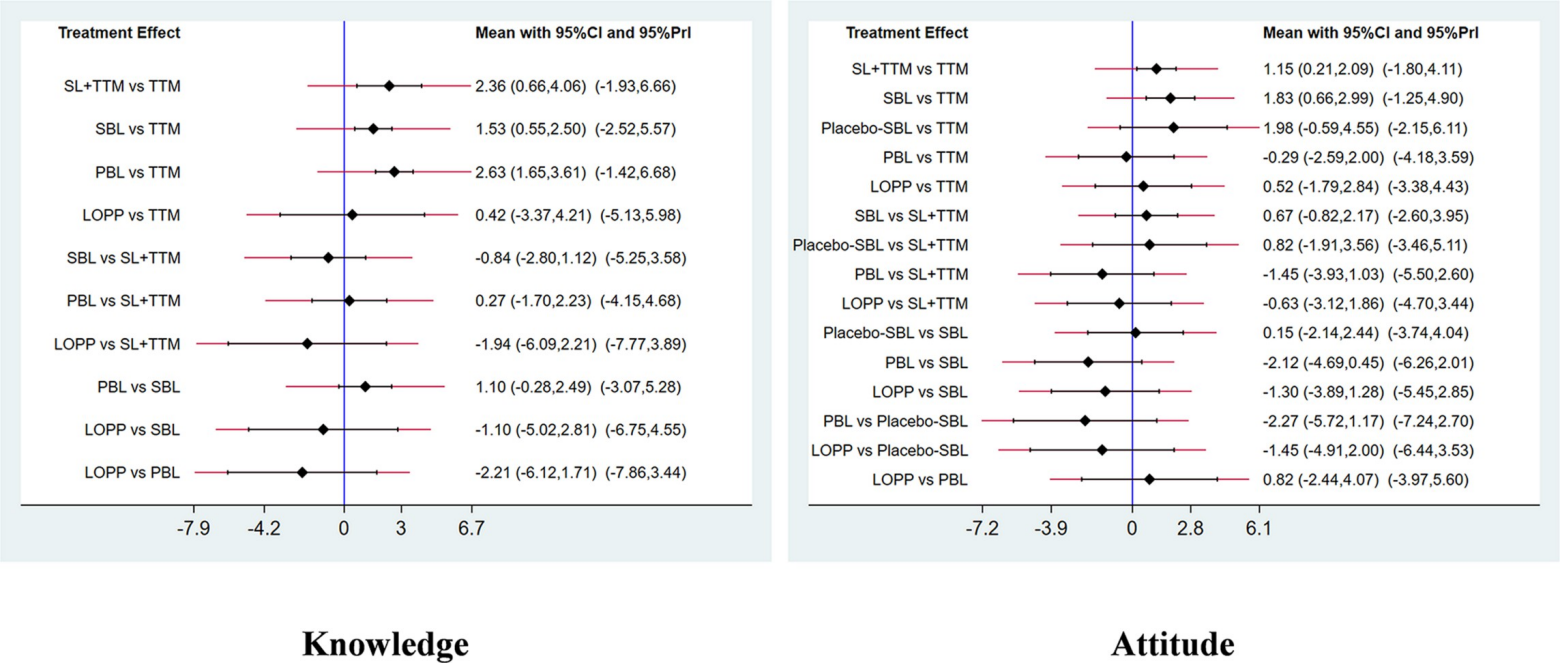
Forest map of the influence of different teaching methods on nursing students’ knowledge and attitude.

36 studies [13, 14, 16, 18, 19, 21–51] provided data on nursing students’ knowledge, involving 5 teaching methods, including SBL, SL + TTM, LOPP, PBL, TTM. The results of network Meta-analysis reveal that the results of inconsistency test were non-significant inconsistency (P > 0.05). Consequently, the adoption of the consistency model for fitting is warranted. Statistical significance is observed in the comparison between SL + TTM, SBL, PBL and traditional teaching method in terms of application effect (P < 0.05), as illustrated in Fig 5.

13 studies [13–25] provided data on nursing students’ attitude toward the elderly, involving 6 teaching methods, including SBL, Placebo SBL, SL + TTM, LOPP, PBL, TTM. The results of network Meta-analysis reveal that the results of inconsistency test were non-significant inconsistency (P > 0.05). Statistical significance is observed in the comparison between SBL, SL + TTM and traditional teaching method in terms of applied effects (P < 0.05), as illustrated in Fig 5.

A higher SUCRA value indicates that the corresponding teaching method yields a more favorable application effect in enhancing the knowledge level of nursing students and their attitude toward the elderly.

**Fig 6:**
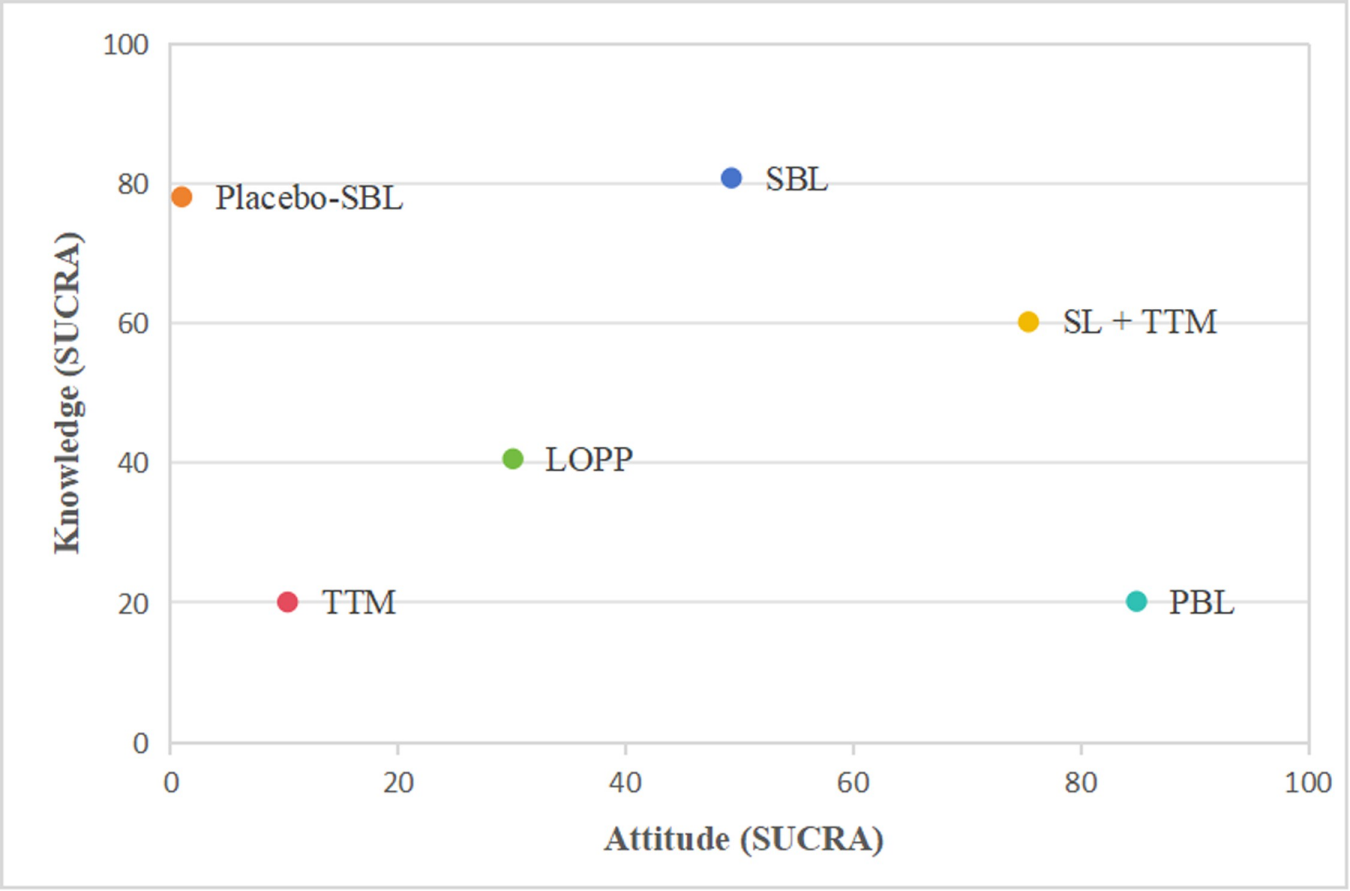
Scatter plot of SUCRA probability analysis on knowledge and attitude of nursing students by different teaching methods.

The ranking for knowledge is as follows: PBL > SL + TTM > SBL > LOPP > TTM; for attitude: SBL > Placebo SBL > SL + TTM > LOPP > PBL > TTM, as shown in Table 2. Additionally, SL + TTM performs the best in terms of the comprehensive aspect of knowledge and attitude, as indicated by the scatter plot (Fig 6) generated from the SUCRA values for knowledge and attitude.

**Table 2:** Different teaching methods of SUCRA sorting table.

Teaching methods	Knowledge	Attitude
SBL	49.8	80.8
Placebo SBL	-	78.1
SL + TTM	76.6	60.2
LOPP	30.3	40.6
PBL	83	20.2
TTM	10.3	20.1

#### Publication bias analysis.

**Fig 7:**
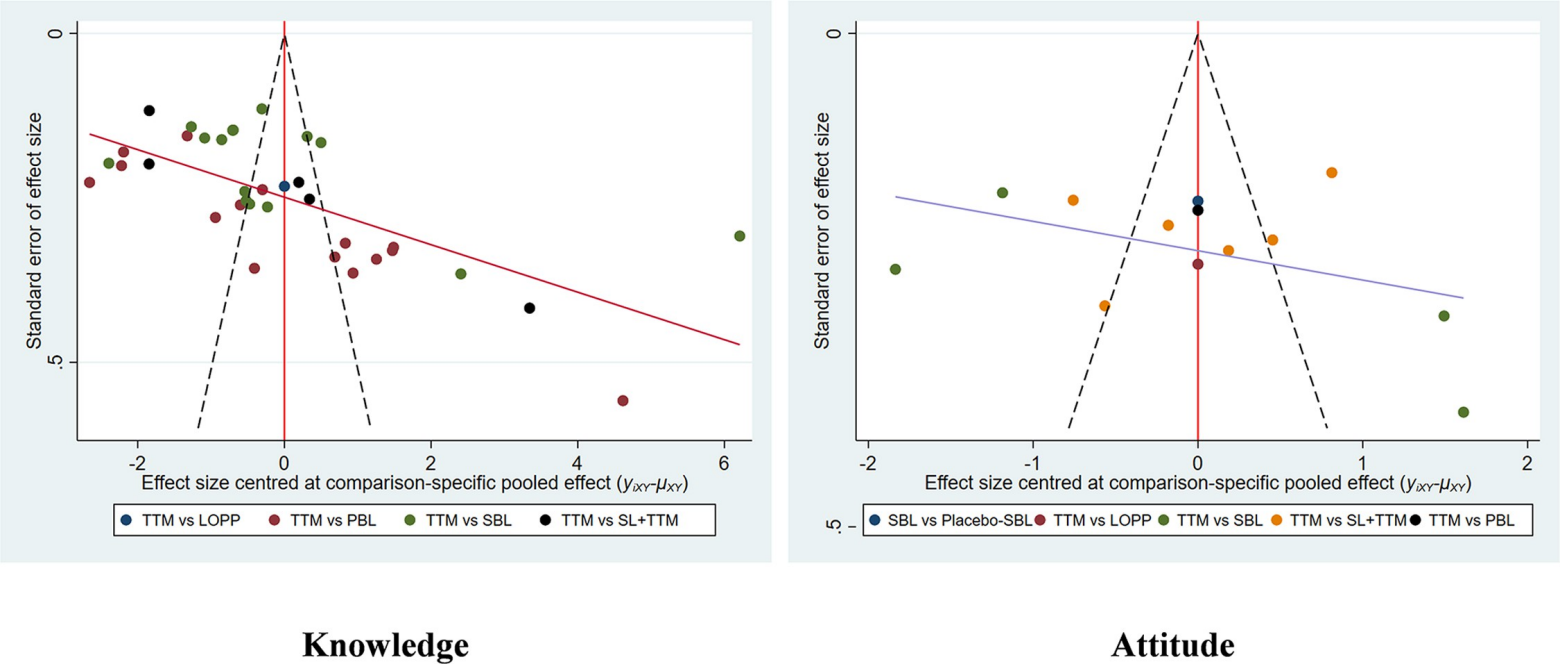
Funnel plot of the influence of different teaching methods on nursing students’ knowledge and attitude.

In terms of knowledge, both Egger’s test (P = 0.001) and an asymmetric funnel plot suggest a potential publication bias. For attitudes, Egger’s test (P = 0.329) and a symmetric funnel plot indicate the absence of publication bias. The funnel plot is shown in Fig 7. Sensitivity analysis was performed by one by one exclusion method, and no significant change was found after the exclusion of any literature, indicating that the results of this study were basically stable.

## Discussion

To the best of our knowledge, this study represents the first attempt to conduct a network meta-analysis of different teaching methods in the context of geriatric nursing education, particularly focusing on the impact of these methods on nursing students’ attitude toward the elderly. The effectiveness of six teaching strategies was evaluated, and literature results were collected from 39 studies involving 5310 nursing students. It was found in results of network meta-analysis that SBL, PBL, and SL+TTM showed statistically significant advantages over TTM in influencing nursing students’ acquisition of knowledge and improvement in attitude, either in single or dual aspects. Specifically, PBL is most effective in enhancing nursing students’ knowledge understanding of geriatric nursing, while SBL demonstrates the best application effect in improving nursing students’ attitude toward the elderly. When considering both indicators simultaneously, SL+TTM is found to exhibit the most optimal comprehensive effectiveness.

Numerous teaching methods have been developed in nursing education to improve the teaching process and enhance the practical effectiveness of nursing education. However, while nursing education has been enriched by these various nursing teaching methods, teachers are burdened in selecting teaching methods, especially when certain learning and usage costs are entailed by these methods. Therefore, evaluating the effectiveness of teaching methods in geriatric nursing education to provide insights into geriatric nursing education is a pressing challenge. Therefore, network meta-analysis was used to examine the effects of teaching methods on nursing students’ knowledge acquisition and attitude toward the elderly, assessing the statistical significance of teaching method comparisons, and analyzing the relative effectiveness of various teaching methods based on SUCRA rankings. Based on the comparative results, teaching methods can be selected by teachers according to teaching objectives to reduce teaching costs and achieve better teaching outcomes.

When the acquisition of theoretical knowledge is used to evaluate the effectiveness of geriatric nursing education, PBL yields the best results. It has been shown by Sayyah et al.’s meta-analysis [52] that it can significantly enhance students’ knowledge levels. This may be because students are actively guided by teachers to learn, actively think, and solve problems together in groups, thereby enhancing their understanding of geriatric nursing knowledge. Furthermore, PBL is widely applied by most educators in the clinical practice teaching of nursing students, promoting students’ comprehension abilities and critical thinking skills [53, 54]. Therefore, PBL enhances nursing students’ ability to analyze and apply geriatric nursing knowledge, leading to effective teaching outcomes.

When attention is dedicated to nursing students’ attitude toward the elderly, SBL emerges as the optimal method. Eost‐Telling et al.’s systematic review [55] indicates that the application of simulation teaching in geriatric nursing education can significantly enhance nursing students’ attitude toward the elderly. This enhancement is primarily attributed to the immersive nature of simulation teaching, wherein nursing students wear elderly simulation suits or other sensory impairment devices, thus simulating and experiencing the sensory limitations typical of the elderly, including visual, auditory, olfactory, thermal, and joint limitations. By actively engaging in activities such as participating in the daily routines of the elderly, assuming the roles of elderly patients, or interacting with the elderly in nursing homes, nursing students are prompted to reflect deeply on their experiences. Consequently, this active reflection fosters the development of positive attitude toward the elderly while simultaneously reducing negative attitude among nursing students.

It is worth noting that SL + TTM performs best when the applying effect in knowledge and attitude are taken into account comprehensively. Through collaboration between schools and communities [56], nursing students are organized to provide services to the elderly in nursing homes or communities with clear learning objectives. SL+TTM allows nursing students to integrate theoretical knowledge with practical application, engage in reflection and discussion, and thereby enhance their understanding of the aging process. Consequently, this method not only improves nursing students’ knowledge levels [57] but also fosters positive attitude toward the elderly [58]. This suggests that SL+TTM is a teaching method worthy of promotion due to its ability to effectively enhance both knowledge acquisition and attitude improvement among nursing students.

Limitations: the methodological quality is relatively low, with 12 articles being quasi-experimental studies and thus possessing a lower level of evidence. Furthermore, some of the teaching methods have limited literature containing outcome indicators related to this study, potentially influencing the results. Therefore, in future research designs, direct comparisons among different teaching methods, supported by RCTs, can be conducted to provide recommended insights into selecting more effective geriatric nursing education models.

## Conclusions

In summary, we conducted a network meta-analysis to study the effects of six teaching methods on improving nursing students’ understanding of elderly care knowledge and their attitude toward the elderly. The results indicate that PBL is most effective in enhancing nursing students’ knowledge of geriatric nursing, while SBL is most effective in improving nursing students’ attitude toward the elderly. When considering both knowledge and attitude, SL + TTM demonstrates the best comprehensive effectiveness. Therefore, educators in geriatric nursing education should prioritize the adoption of these teaching methods to enhance nursing students’ knowledge and improve attitude.

## Supporting information

S1 Table The PRISMA network meta-analysis checklist. (DOCX)

S2 Table The initial search strategy for PubMed. (DOCX)
