# Supplementary material for: Effects of different geriatric nursing teaching methods on nursing students’ knowledge and attitude: Systematic review and network meta-analysis
Source: PLoS One. 2024 May 31;19(5):e0300618. doi: 10.1371/journal.pone.0300618 (PMC11142439; doi:10.1371/journal.pone.0300618)
Supplement: S2 Table — (DOCX) [file pone.0300618.s002.docx]

**S2 Table. The initial search strategy for PubMed**

| ****Steps**** | ****Search**** |
| --- | --- |
| **#1** | **(((((((Students, Nursing[MeSH Terms]) OR (Students, Nursing[Title/Abstract])) OR (Nursing Students[Title/Abstract])) OR (Nursing Student[Title/Abstract])) OR (Pupil Nurses[Title/Abstract])) OR (Nurses, Pupil[Title/Abstract])) OR (Nurse, Pupil[Title/Abstract])) OR (Pupil Nurse[Title/Abstract])** |
| **#2** | **(((((Geriatric nursing[Title/Abstract]) OR (Gerontology[Title/Abstract])) OR (Curriculum of Gerontological nursing[Title/Abstract])) OR (Education[Title/Abstract])) OR (Gerontological nursing education[Title/Abstract])) OR (Geriatric[Title/Abstract])** |
| **#3** | **(((((((((((((((Simulation-based learning[Title/Abstract]) OR (SBL[Title/Abstract])) OR (Simulation-based education[Title/Abstract])) OR (SBE[Title/Abstract])) OR (Simulation[Title/Abstract])) OR (Experiential learning[Title/Abstract])) OR (Experiential teaching[Title/Abstract])) OR (Service learning[Title/Abstract])) OR (Problem-based learning[Title/Abstract])) OR (PBL[Title/Abstract])) OR (lecture-based learning[Title/Abstract])) OR (LBL[Title/Abstract])) OR (Learning with older people programme[Title/Abstract])) OR (LOPP[Title/Abstract])) OR (traditional teaching[Title/Abstract])) OR (TTM[Title/Abstract])** |
| **#4** | **(((((((((((((((((((((((((((((((((((((((((((knowledge[Title/Abstract]) OR (final examination scores[Title/Abstract])) OR (final course scores[Title/Abstract])) OR (test scores[Title/Abstract])) OR (school marks[Title/Abstract])) OR (scholastic achievement[Title/Abstract])) OR (theoretical knowledge[Title/Abstract])) OR (academic performance[Title/Abstract])) OR (course grade[Title/Abstract])) OR (exam scores[Title/Abstract])) OR (final exam scores[Title/Abstract])) OR (Attitudes towards older[Title/Abstract])) OR (Attitudes towards elderly[Title/Abstract])) OR (Attitudes towards geriatrics[Title/Abstract])) OR (Attitudes towards old[Title/Abstract])) OR (Attitudes towards aged[Title/Abstract])) OR (Attitudes towards aging[Title/Abstract])) OR (Attitudes towards seniors[Title/Abstract])) OR (Attitude towards older[Title/Abstract]) OR (Attitude towards elderly[Title/Abstract])) OR (Attitude towards geriatrics[Title/Abstract])) OR (Attitude towards old[Title/Abstract])) OR (Attitude towards aged[Title/Abstract])) OR (Attitude towards aging[Title/Abstract])) OR (Attitude towards seniors[Title/Abstract])) OR (Attitude towards older[Title/Abstract]) OR (Attitudes to elderly[Title/Abstract])) OR (Attitudes to geriatrics[Title/Abstract])) OR (Attitudes to old[Title/Abstract])) OR (Attitudes to aged[Title/Abstract])) OR (Attitudes to aging[Title/Abstract])) OR (Attitudes to seniors[Title/Abstract])) OR (Attitude to older[Title/Abstract]) OR (Attitude to elderly[Title/Abstract])) OR (Attitude to geriatrics[Title/Abstract])) OR (Attitude to old[Title/Abstract])) OR (Attitude to aged[Title/Abstract])) OR (Attitude to aging[Title/Abstract])) OR (Attitude to seniors[Title/Abstract])) OR (ageism[Title/Abstract])) OR (prejudice[Title/Abstract])) OR (discrimination[Title/Abstract])) OR (stereotyping[Title/Abstract])) OR (stigma[Title/Abstract])** |
| **#5** | **((((((randomized controlled trial) OR (randomization)) OR (RCT)) OR (controlled clinical trail)) OR (CCT)) OR (quasi-experimental study)) OR (experimental study)** |
| **#6** | **#1 AND #2 AND #3 AND #4 AND #5** |
